# Severe Chest Wall Toxicity From Cryoablation in the Setting of Prior Stereotactic Ablative Radiotherapy

**DOI:** 10.7759/cureus.477

**Published:** 2016-02-02

**Authors:** Aadel A Chaudhuri, Michael S Binkley, Sonya Aggarwal, Yushen Qian, Justin N Carter, Rajesh Shah, Billy W Loo Jr.

**Affiliations:** 1 Department of Radiation Oncology, Stanford University School of Medicine; 2 Department of Interventional Radiology, Stanford University School of Medicine; 3 Stanford Cancer Institute

**Keywords:** stereotactic ablative radiotherapy (sabr), stereotactic body radiation therapy, cryoablation, cryoablation toxicity, radiation related toxicities, chest wall necrosis, synovial sarcoma, metastatic cancer, oligometastasis, interventional oncology

## Abstract

We present the case of a 42-year-old woman with metastatic synovial sarcoma of parotid origin, treated definitively with chemoradiation, who subsequently developed oligometastatic disease limited to the lungs. She underwent multiple left and right lung wedge resections and left lower lobectomy, followed by right lower lobe stereotactic ablative radiotherapy (SABR), 54 Gy in three fractions to a right lower lobe lesion abutting the chest wall. Two years later, she was treated with cryoablation for a separate right upper lobe nodule abutting the chest wall. Two months later, she presented with acute shortness of breath, pleuritic chest pain, decreased peripheral blood O2 saturation, and productive cough. A computed tomography (CT) scan demonstrated severe chest wall necrosis in the area of recent cryoablation that, in retrospect, also received a significant radiation dose from her prior SABR. This case demonstrates that clinicians should exercise caution in using cryoablation when treating lung tumors abutting a previously irradiated chest wall.

Note: Drs. Loo and Shah contributed equally as co-senior authors.

## Introduction

As systemic cancer therapies continue to improve, oncologists are seeing more patients with oligometastatic or oligoprogressive disease, where metastatic disease is well controlled except for in discrete locations. For these cases, we have become more comfortable offering focal therapies, such as surgery, stereotactic ablative radiotherapy (SABR), and/or cryoablation. However, the risk of multimodal local tumor therapy is not fully understood in these scenarios. It is also not always well understood how much time and distance is safe between different treatment modalities. Here, we describe a case of a patient who developed severe chest wall necrosis after cryoablation in the setting of prior SABR to a nearby chest wall-abutting nodule. Informed consent was obtained from the patient for this study.

## Case presentation

**Figure 1:**
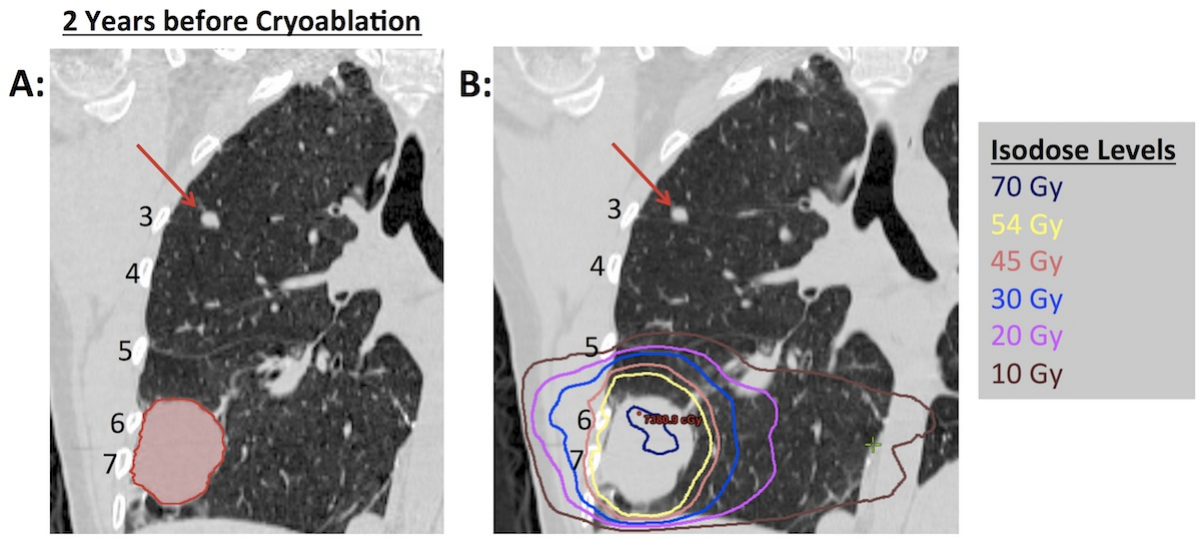
Right lower lobe nodule abutting the chest wall treated with SABR (54 Gy in three fractions) (A) Right lower lobe nodule highlighted in red. (B) SABR treatment plan with radiotherapy dose depicted as isodose levels. Red arrows indicate right upper lobe nodule subsequently treated two years later with cryoablation. Rib numbers are as indicated. SABR = stereotactic ablative radiotherapy.

A 40-year-old female with a synovial sarcoma of parotid origin developed lung-only oligometastases 17 months after completing her original course of definitive therapy. She underwent chemotherapy, bilateral lung wedge resections, and a left lower lobectomy. Six years after the diagnosis, she presented to our clinic for consideration of stereotactic ablative radiotherapy (SABR) of a right lower lobe oligometastasis abutting the lateral sixth rib. The lesion was round, well-defined, and abutted the right chest wall next to the sixth and seventh ribs (Figure 1A). We felt that she was a good candidate for lung SABR, and treated her with 54 Gy in three fractions using image-guided radiotherapy and inspiratory breath-hold technique (Figure 1B). The chest wall at the level of the lateral fifth rib received 10-30 Gy (Figure 1B).

**Figure 2:**
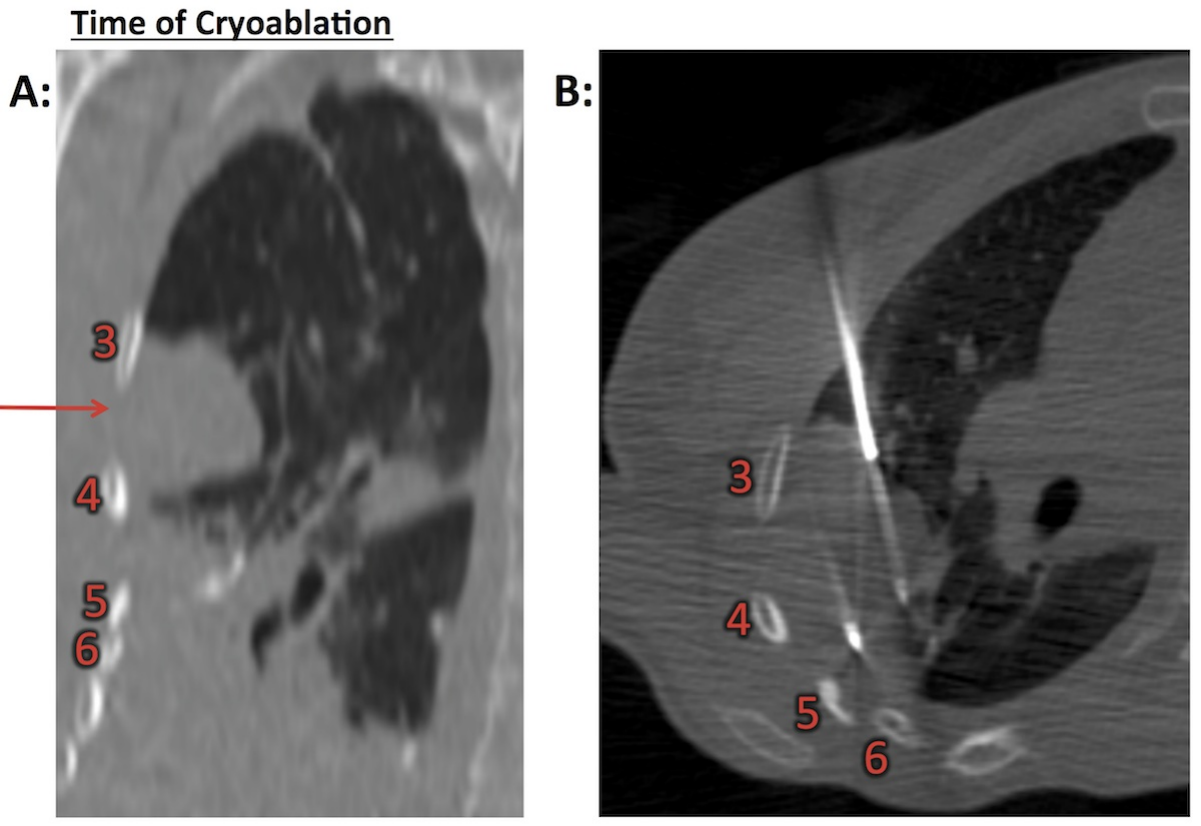
Right upper lobe nodule abutting the chest wall treated with cryoablation (A) Right upper lobe nodule indicated by red arrow. (B) Axial image with cryoablation probes in place. Rib numbers are as indicated. In retrospect, the right upper lobe nodule that was originally distant from the prior radiation field is now adjacent to a portion of the chest wall that received significant radiation dose because of interval right lower lobe volume loss.

Two years after SABR, she developed progression of a separate oligometastatic right upper lung nodule. This had been present two years earlier (Figure 1A), originally well superior to the lesion previously treated with SABR, but had grown substantially in the interval and was now abutting the right lateral chest wall (Figure 2A). Because of concerns of cumulative lung parenchymal damage, given her prior course of SABR and multiple surgical resections, she was referred to interventional radiology for consideration of a complementary modality to provide local tumor control. Cryoablation was administered, and the tumor was treated with six cryoprobes with three freeze cycles totaling 20 minutes (Figure 2B).

During the procedure, the patient developed hypoxia with SpO_2_ 89%, requiring intubation. A CT post-procedure showed extensive hemorrhage involving all lobes of the right lung. The patient recovered after a short ICU stay and was discharged home in stable condition on 1.5-2 L nasal cannula oxygen.

**Figure 3:**
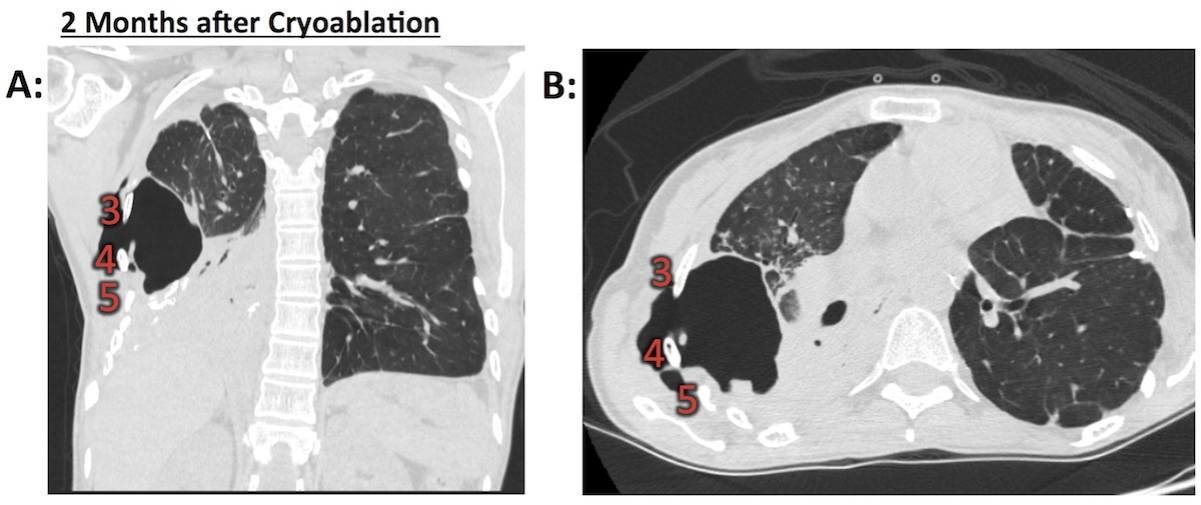
Severe right chest wall toxicity two months following cryoablation A) Coronal and B) Axial views of right-sided cavitary lesion extending through the chest wall past the ribs, with substantial soft tissue destruction and adjacent right lower lobe dense consolidation. Rib numbers are as indicated.

Two months later, the patient was admitted to the hospital with four days of fever, productive cough, shortness of breath, and right-sided pleuritic chest pain. A CT demonstrated a large cavitary lesion, extending from the right peripheral lung through the adjacent right lateral chest wall at the level of ribs three, four, and five into the musculature, with adjacent lobar consolidation (Figure 3).

We monitored the patient closely on telemetry, and administered broad-spectrum antibiotics, nasal cannula oxygen, nebulizers, and chest physical therapy. Subsequent CT scans showed the persistence of the large right lung cavitary lesion extending through the right lateral chest wall, but an improvement of right-sided lobar consolidation. Her respiratory symptoms improved, she became afebrile and was discharged from the hospital in stable condition after a five-day inpatient stay, with her previous baseline requirement of 1.5-2 L nasal cannula oxygen. We considered surgical repair of the large chest wall defect, but with substantial concerns about impaired healing due to prior radiotherapy and cryotherapy. She declined surgery in favor of supportive care. Her respiratory symptoms ultimately worsened, and she died six months later. 

## Discussion

Studies have shown that SABR of lung tumors near the chest wall can be associated with toxicity, such as chest wall pain and rib fractures [1-2]. However, severe toxicity, such as chest wall necrosis, is extremely rare, limited to a case report by Woody, et al. of a case of chest wall abscess and bronchopleural fistula 17 months following SABR (50 Gy in five fractions) of a pleural-based left lower lobe non-small cell lung cancer [3]. Factors contributing to chest wall toxicity following SABR include tumor size, tumor location, treatment volume, chest wall radiation dose, and potentially bone density and body habitus [2, 4-6].

Similarly, severe chest wall toxicity after cryoablation, even of tumors abutting the chest wall, is extremely rare [7]. Cryoablation has been shown in isolated cases to cause irreversible thermal damage [8]. Ito, et al. showed in their retrospective cohort that 35% of lung cryoablation zones cavitate after treatment, but that 96% of these resolve by six months [9].

In our patient’s case, since we treated a right lower lobe oligometastasis directly abutting the chest wall with SABR 54 Gy, we elected to use a complementary modality to treat a separate right upper lobe nodule two years later. The new nodule was treated with cryoablation, which was followed by severe chest wall toxicity, including massive soft tissue necrosis. In retrospect, although the right upper lobe lesion treated with cryotherapy was originally far outside the treatment field of the right lower lobe lesion treated with SABR, over time, post-SABR fibrosis and volume loss led to the right upper lobe lesion moving inferiorly and abutting the region of the chest wall within the prior radiation field at the level of ribs five and six (Figure 2). We hypothesize that the combination of cryotherapy involving a previously irradiated area of chest wall led to the development of unexpected severe chest wall toxicity. This phenomenon has not been previously reported per our review of the literature.

There is no standard set of dosimetric or cryotherapy constraints when using these complementary modalities. Thus, it is difficult to predict whether a patient might experience a severe toxicity as our patient unfortunately did. However, it is well-known that radiotherapy impairs wound healing. This case illustrates that we should exercise caution when performing cryoablation in the setting of prior SABR for lesions abutting the chest wall. We cannot exclude the possibility that cryoablation overlapping with less intensive palliative radiotherapy doses could also be toxic.

## Conclusions

We present here a report of severe chest wall necrosis following cryoablation of a nodule abutting a portion of chest wall that received a significant dose of radiation from a prior course of stereotactic ablative radiotherapy to a separate lesion. We recommend caution when combining these two modalities for treatment of pulmonary tumors abutting the chest wall.
